# The liver Metastatic Adenocarcinoma of Colorectal Cancer With Synchronous Isolated Hepatic Tuberculosis

**DOI:** 10.5812/hepatmon.9844

**Published:** 2013-05-16

**Authors:** Mohammadreza Hafezi Ahmadi, Hadi Teimouri, Sajjad Alizadeh

**Affiliations:** 1 Department of Pathology, Ilam University of Medical Sciences, Ilam, IR Iran; 2 Student Research Committee, Ilam University of Medical Sciences, Ilam, IR Iran

**Keywords:** Tuberculosis, Adenocarcinoma, Neoplasm Metastasis

*Dear Editor,*

Tuberculosis is a global problem. According to the World Health Organization, more than 2 billion people are currently infected with tuberculosis bacilli and 1.7 million deaths attributed to TB in 2006 (1). TB usually occurs in lung and other form of it like primary abdominal TB is not uncommon and 6-38% of patients with active pulmonary TB have abdominal TB (2). Hepatic tuberculosis is usually associated with military tuberculosis or active pulmonary and isolated liver tuberculosis (ILT) is a rare type of TB (3, 4). Hereby we report a case of isolated hepatic tuberculosis. A 54 year old Iranian man has suffered from gastrointestinal symptoms, changes in bowel habits and rectal bleeding and abdominal pain for 3 month. Laboratory data revealed hematocrit 33%; hemoglobin 10.2 g/dL; white blood cells 9400/mm³ (71% neutrophils). Liver enzymes showed: total bilirubin of 9 mg/L with a direct bilirubin of 4.3 mg/L; SGOT and SGPT were 138 U/L and 192 U/L respectively (normal range for SGOT is 17-59 U/L and for SGPT is 21-72 U/L). Abdominal examination was unremarkable except for mild tenderness in RUQ. Patients had not history of colon cancer or TB in familial history. The patient was undergoing colonoscopy, a lesion measuring 3.0 × 4.1 × 1.5 cm in the sigmoid colon was found. The biopsy of the lesion was referred to the lab. Colon adenocarcinoma diagnosed before surgery. For metastatic work up, the patient undergoing CT and a lesion in the right lobe of the liver was observed which a biopsy was taken from. Our initial diagnosis according to the patient with adenocarcinoma and colonoscopy results, before operation, and the finding of patients CT were metastatic lesion. For the final diagnosis of the lesion we used biopsy and pathologic findings which were positive for tuberculosis and metastatic adenocarcinoma in liver. Lung was cleared for tuberculosis in studies and isolated hepatic tuberculosis diagnosed. However TB can diffuse in other organ like mesenteric lymph nodes, gastrointestinal tract and Urinary tract but isolated hepatic tuberculosis is rare condition. Overall we know 3 forms of hepatic tuberculosis. Diffuse hepatic with pulmonary or miliary tuberculosis, which is the most common form. Diffuse hepatic infiltration without recognizable pulmonary involvement and third form usually presents as a focal – local or abscess. This form is very rare (5, 6). In Kok KY study, Incidence of isolated hepatic tuberculosis estimated as0.3% (7). Many studies confirm that Clinical presentation of isolated hepatic tuberculosis is so atypical and we can`t only use imaging for diagnosing the ILT, because many other of focal lesions in liver, such as metastases, hepatocellular carcinoma are similar to that, so pathology examination is necessary for distinguishing tuberculosis from lymphoproliferative disorder and other granulomatous diseases (7-9). Usually Pathologic finding in tuberculosis liver is a necrotizing and granolomatus. likely gaint cells are found in the granuloma which are surrounded by lymphohistiocytic cells, plasma cells and eosinophils (10). Unfortunately, in most of cases the diagnosis of liver TB is made only after exploratory laboratory or biopsy, as the present case. Therefore the diagnosis of liver TB should be considered in the context of a mass in the right lobe of the liver in patients from endemic TB zone or in the immunocompromised patients.

**Figure 1.**
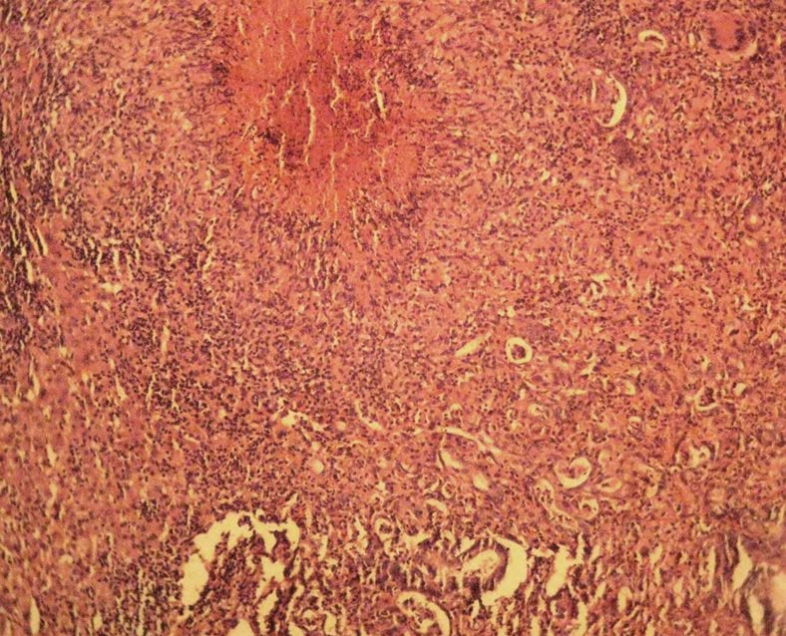
Images of a Necrosis Granulomatous in Biopsy of Liver and Pleomorphic Tumor Cells are Hyperchromatic With Irregular Nuclear Borders (× 400 H&E stain)
